# The Impact of IgG Glycosylation in SARS-CoV-2 Infection vs. Vaccination: A Statistical Analysis

**DOI:** 10.3390/ijms27020946

**Published:** 2026-01-18

**Authors:** Adriána Kutás, Attila Garami, Csaba Váradi

**Affiliations:** 1 Materials and Intelligent Technologies Higher Education and Industrial Cooperation Centre, University of Miskolc, 3515 Miskolc, Hungary; kutas.adriana@student.uni-miskolc.hu; 2 Institute of Energy, Ceramic and Polymer Technology, University of Miskolc, 3515 Miskolc, Hungary; attila.garami@uni-miskolc.hu; 3 Institute of Chemistry, Faculty of Materials Science and Chemical Engineering, University of Miskolc, 3515 Miskolc, Hungary

**Keywords:** IgG glycosylation, SARS-CoV-2, COVID-19 vaccination, sialylated glycans, high mannose glycans, biomarkers

## Abstract

This study investigates the glycosylation patterns of serum IgG antibodies in relation to COVID-19 infection and vaccination, highlighting the potential of specific glycan profiles as biomarkers for immune responses. Using Spearman correlation analysis, distinct associations among glycan levels and various clinical laboratory parameters were identified, revealing complex, non-linear interactions that influence immune dynamics. Significant differences were observed in sialylated glycan profiles across patient groups, indicating that vaccination and natural infection elicit unique immune mechanisms and suggesting that vaccination induces favorable glycosylation changes. Notably, high-mannose glycans were found to correlate with other glycan types, underscoring their critical role in the immune response and suggesting their potential as biomarkers to differentiate between infection- and vaccination-induced immunity. The findings suggest that understanding these glycosylation dynamics may enhance diagnostic and therapeutic strategies, providing valuable tools for differentiating between immune responses elicited by infection and vaccination. Overall, this study contributes to the understanding of glycosylation’s impact on immune function in the context of COVID-19, emphasizing the importance of specific glycan markers, such as sialylated and high-mannose structures, in clinical applications.

## 1. Introduction

The COVID-19 pandemic has prompted extensive research into the immune responses elicited by both natural infection and vaccination. Understanding these responses remains critical for developing effective diagnostic tools and therapeutic strategies, as the landscape of COVID-19 continues to evolve [1]. SARS-CoV-2 has revealed the complexities of the human immune system and the need for a nuanced understanding of how it reacts to different forms of exposure [2].

One area of particular interest is the role of glycosylation patterns in serum IgG antibodies, which are essential components of the adaptive immune response [3]. Glycosylation, the biochemical process by which carbohydrate structures (glycans) are attached to proteins, plays a pivotal role in modulating antibody function, stability, and overall immune efficacy [4]. The glycosylation of antibodies can influence their ability to bind to antigens, activate complement pathways, and mediate interactions with immune cells [5]. Recent studies have indicated that variations in glycan structures can significantly affect the immune response to pathogens, thereby impacting disease outcomes [6].

Emerging evidence suggests that the glycosylation patterns of antibodies may vary significantly depending on the individual’s history of infection or vaccination [7]. For instance, vaccinated individuals may exhibit distinct glycan profiles compared to those who have recovered from COVID-19, suggesting that the immune system’s memory and response mechanisms are different between the two groups [8]. This raises the possibility that specific glycan markers could be leveraged as biomarkers for differentiating between immune responses, with implications for monitoring vaccine efficacy and disease progression [9].

This study aims to investigate the glycosylation patterns of serum IgG antibodies among individuals with varying COVID-19 infection and vaccination histories. By analyzing the correlations among glycan levels and various laboratory parameters, we seek to uncover complex interactions that may influence immune dynamics. A particular emphasis will be placed on the role of sialylated glycans, as preliminary findings suggest that these structures play a critical role in modulating antibody functionality and overall immune response. Additionally, the study will explore the interactions of high-mannose glycans with other glycan types, as their interconnectedness may reveal important insights into the immune response. High-mannose glycans have been implicated in various immune processes, and understanding their roles could enhance our knowledge of how the immune system responds to viral infections.

In summary, this study contributes to the growing body of evidence regarding the impact of glycosylation on immune function in the context of COVID-19. By elucidating the relationships between glycan profiles and immune responses, we aim to enhance the understanding of immune dynamics and inform future diagnostic and therapeutic strategies. Ultimately, these insights could lead to more effective interventions for managing COVID-19 and improving patient outcomes, thereby addressing a critical need in public health.

## 2. Results

The study reveals several significant trends regarding the glycosylation patterns of serum IgG antibodies in the context of COVID-19 infection and vaccination, as illustrated in the accompanying figures.

**Figure 1:**
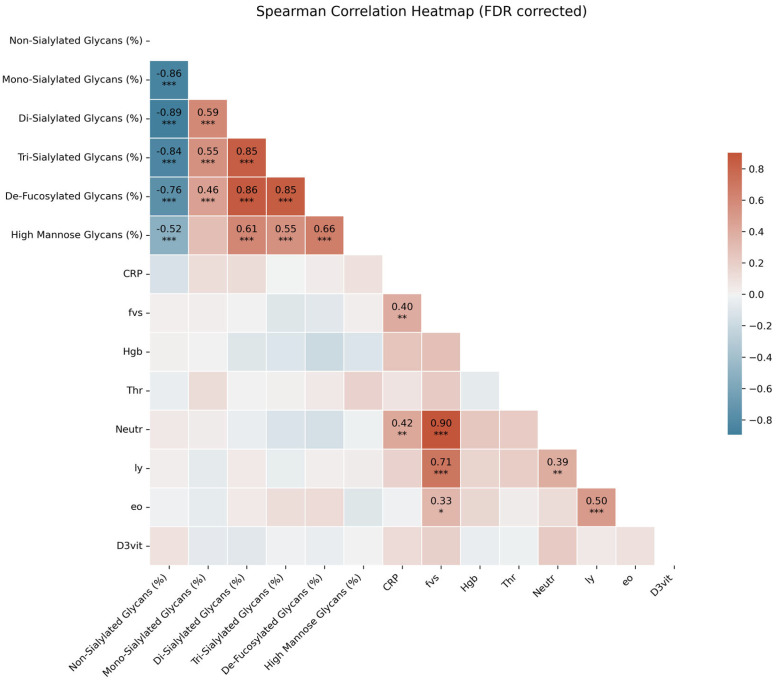
Spearman correlation matrix illustrating the relationships between serum IgG glycan groups and clinical laboratory parameters across the study cohorts (N = 64). The color scale represents the Spearman correlation coefficient, ranging from red (positive correlation) to blue (negative correlation). *p*-values indicating statistical significance are annotated within each cell; values where *p* < 0.05 denote significant associations between glycosylation profiles and systemic physiological markers (* *p* < 0.05, ** *p* < 0.01, *** *p* < 0.001).

The correlation analysis, as shown in Figure 1, reveals both positive and negative associations among glycan levels and various laboratory parameters. This Spearman correlation matrix illustrates complex interactions that may influence immune dynamics, indicating that certain glycan levels are linked in ways that could affect how the immune system responds to the virus. The presence of nonlinear relationships points to the idea that these interactions are not straightforward, suggesting that multiple factors, including individual patient characteristics and clinical markers, may play a role in shaping these relationships.

**Figure 2:**
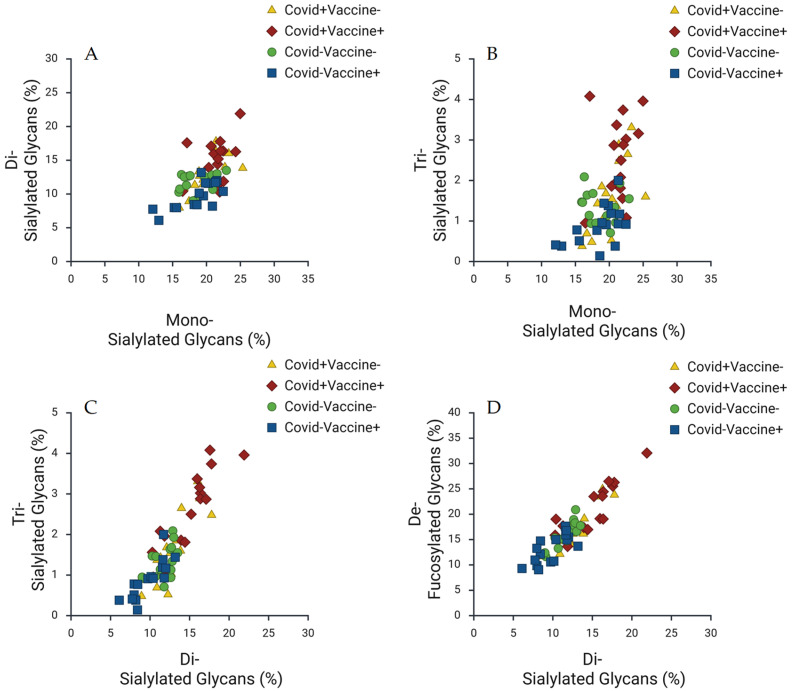
Correlation of IgG Glycan Profiles by COVID-19 Infection and Vaccination Status. Scatter plots depicting the relationships between different types of sialylated glycans in serum IgG antibodies across various COVID-19 infection and vaccination groups. (**A**): Di-sialylated Glycans (%) vs. Mono-sialylated Glycans (%). (**B**): Tri-sialylated Glycans (%) vs. Mono-sialylated Glycans (%). (**C**): Tri-sialylated Glycans (%) vs. Di-sialylated Glycans (%). (**D**): De-fucosylated Glycans (%) vs. Di-sialylated Glycans (%). Data points represent different patient groups: Yellow triangles indicate COVID+Vaccine−, red diamonds denote COVID+Vaccine+, green circles represent COVID−Vaccine−, and blue squares illustrate COVID−Vaccine+. Each plot highlights significant variations in glycan profiles, demonstrating the impact of COVID-19 infection and vaccination on IgG glycosylation dynamics.

One of the primary observations made is the emergence of distinct glycan profiles based on individuals’ COVID-19 infection and vaccination status. This trend is highlighted in Figure 2, which presents scatter plots that depict variations in sialylated glycan types across different patient groups. It is suggested that vaccination and natural infection elicit unique immune response mechanisms, leading to varied glycosylation patterns. For instance, the distinct glycan profiles associated with vaccinated individuals compared to those who have been infected underscore the potential for these profiles to serve as biomarkers for immune responses.

**Figure 3:**
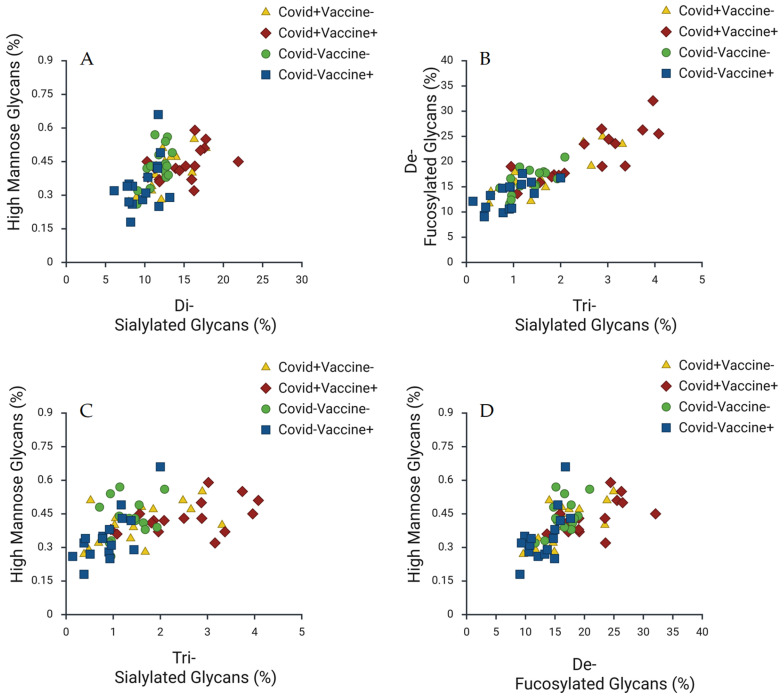
Correlation of High-Mannose Glycans with Other Glycan Types by COVID-19 Infection and Vaccination Status. Scatter plots illustrating the relationships between high-mannose glycan percentages and other glycan types in serum IgG antibodies across different COVID-19 infection and vaccination groups. (**A**): High-Mannose Glycans (%) vs. Di-sialylated Glycans (%). (**B**): De-fucosylated Glycans (%) vs. Tri-sialylated Glycans (%). (**C**): High-Mannose Glycans (%) vs. Tri-sialylated Glycans (%). (**D**): High-Mannose Glycans (%) vs. De-fucosylated Glycans (%). Data points represent different patient groups: yellow triangles indicate COVID+Vaccine−, red diamonds denote COVID+Vaccine+, green circles represent COVID−Vaccine−, and blue squares illustrate COVID−Vaccine+.

Further examination of the dynamics of sialylated glycans, as depicted in Figure 2, provides insights into how different types of sialylated glycans (e.g., di-sialylated and tri-sialylated) correlate with one another across patient groups. The variations in sialylation levels reinforce the notion that both infection and vaccination significantly impact the glycosylation of IgG antibodies, revealing a nuanced understanding of how these glycans function within the immune response. This complexity emphasizes the need to consider the broader context of immune interactions when interpreting glycan profiles. Moreover, Figure 3 explores the relationships between high-mannose glycans and other glycan types, revealing important interactions that suggest high-mannose glycans play a critical role in immune responses. The scatter plots in this figure indicate that these structures correlate with various other glycan types, further underscoring the interconnectedness of glycosylation dynamics. It is proposed that high-mannose glycans could act as potential biomarkers for distinguishing between immune responses elicited by infection versus vaccination, which is crucial for clinical diagnostics. Collectively, these trends highlight the significant impact of both COVID-19 infection and vaccination on IgG glycosylation patterns. The distinct profiles that emerge for each condition suggest that specific glycan markers could be leveraged to effectively differentiate between immune responses. This has important implications for improving diagnostic and therapeutic strategies in managing COVID-19, as it is believed that understanding these glycosylation dynamics may enable better monitoring of immune responses and inform vaccination strategies.

**Figure 4:**
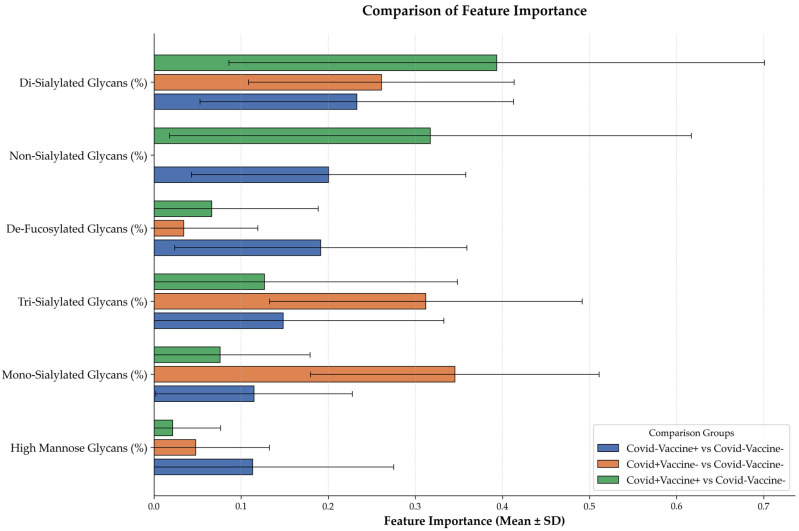
Comparison of Feature Importance of Glycan Types. Di-Sialylated Glycans (green): Highest importance in distinguishing the COVID+Vaccine+ group from the COVID−Vaccine− group, indicating a significant role in eliciting immune responses. Non-Sialylated Glycans (blue): Significant importance in the COVID−Vaccine+ group, highlighting their contribution to immune dynamics. Tri-Sialylated Glycans (orange): Demonstrate notable importance across groups, supporting the notion of distinct immune mechanisms elicited by vaccination. High-mannose Glycans (notable lower importance): Although they exhibit lower feature importance, their correlations with other glycan types suggest potential as biomarkers for differentiating immune responses.

In addition to the observed glycosylation patterns, our analysis of feature importance, as illustrated in Figure 4, further elucidates the role of specific glycan types in distinguishing between immune responses elicited by COVID-19 vaccination and natural infection. The analysis revealed that Di-Sialylated glycans exhibited the highest feature importance, particularly in the comparison of the COVID+Vaccine+ group against the COVID−Vaccine− group. This finding aligns with the hypothesis that sialylated glycans play a pivotal role in enhancing immune responses, as suggested by existing literature emphasizing their involvement in modulating antibody functions. Furthermore, significant feature importance was also noted for Non-Sialylated and Tri-Sialylated glycans, reinforcing the idea that these glycan types contribute meaningfully to the immune dynamics observed in vaccinated individuals. This is consistent with previous studies that have highlighted the distinct glycan profiles associated with vaccination compared to those resulting from natural infection. Interestingly, high-mannose glycans were found to correlate with various other glycan types, suggesting their critical role in the immune response landscape. Although they exhibited lower feature importance compared to sialylated glycans, their interconnectedness with other glycans indicates that they may still serve as valuable biomarkers for differentiating between infection- and vaccination-induced immunity. Collectively, these findings underscore the complex interplay of glycosylation patterns influenced by both COVID-19 infection and vaccination. The distinct glycan profiles emerging from this analysis highlight the potential for specific glycan markers to serve as biomarkers for immune responses, aligning with our earlier discussions on the importance of glycan profiling in clinical diagnostics and therapeutic strategies. This comprehensive understanding of glycosylation dynamics may enhance our approaches in managing COVID-19, providing critical insights into optimizing vaccine efficacy and patient outcomes.

In conclusion, the study illustrates a complex interplay of glycosylation patterns influenced by COVID-19 infection and vaccination, with distinct profiles emerging for each condition. The findings underscore the potential for specific glycan markers to serve as valuable tools for differentiating between immune responses and enhancing clinical strategies for managing COVID-19.

## 3. Discussion

Our finding of distinct glycan profiles associated with individuals’ vaccination and natural infection histories is a key contribution that corroborates existing scientific literature regarding the differential immune responses elicited by these two events. The observed differences, particularly within the sialylated glycan profiles (as shown in Figure 2), strongly suggest that the immune response mechanisms activated are unique. The observed variations in IgG N-glycosylation between infected and vaccinated cohorts are not merely structural; they dictate the effector functions of the humoral immune response. Specifically, the degree of terminal sialylation on the Fc-glycan is a known ‘switch’ for anti-inflammatory activity [10]. Sialylated IgGs exhibit reduced affinity for activating FcγRs and increased binding to inhibitory receptors like FcγRIIB or lectins such as DC-SIGN, thereby raising the threshold for innate immune activation [11,12]. This is particularly relevant in the context of post-vaccination profiles where a shift toward higher sialylation may reflect a stabilized, non-pathogenic immune state [5].

Furthermore, the status of core fucosylation significantly modulates antibody-dependent cellular cytotoxicity (ADCC). The absence of fucose (afucosylation), which we observed as a differentiating factor in our correlation matrix (Figure 1), enhances the affinity of IgG1 for FcγRIIIa by up to 50-fold. This phenomenon is frequently observed in severe COVID-19 cases where it can trigger a ‘cytokine storm’ via alveolar macrophages. Finally, galactosylation levels influence the structural stability of the CH2 domain and its ability to initiate the classical complement pathway (CDC), with low galactosylation (G0) serving as a hallmark of pro-inflammatory conditions. By mapping these specific glycan traits, we provide a molecular framework for understanding the divergent immune trajectories following infection versus immunization [13].

This supports the notion put forth by studies such as that by Assis et al. (2021) that the specific immune response elicited by vaccination differs mechanistically from that of natural infection [14].

The strong correlations identified for high-mannose glycans (Figure 3) underscore their critical and interconnected role within the overall glycosylation dynamic. High-mannose structures have been implicated in various immune processes, particularly in the context of viral infections. Their correlation with other glycan types suggests an intricate, non-random network of glycosylation that is integral to immune modulation. Furthermore, the ability of these glycans to clearly distinguish between the infection and vaccination groups reinforces their potential as novel biomarkers for differentiating between the immune responses elicited by these two events. This differentiation capability holds significant promise for clinical diagnostics, allowing for a clearer, molecular-level assessment of a patient’s immune status and memory.

Moreover, the Spearman correlation analysis (Figure 1), which revealed complex, nonlinear interactions between glycan levels and various clinical laboratory parameters (such as CRP, Hgb, and Vitamin D3 levels), aligns with studies that emphasized the systemic and integrated nature of glycosylation dynamics [15]. These findings highlight that glycosylation is not an isolated process but is significantly shaped by broader physiological factors, including patient demographics, disease severity, and general health markers. This interconnectedness underscores the need for individualized and holistic approaches when interpreting glycan profiles for clinical use, suggesting that integrating both glycan and clinical markers is necessary for accurate diagnosis and prognosis.

The interaction of various glycan types within the immune response is complex and multifaceted. Glycosylation can modulate the binding affinity of antibodies to antigens and influence the activation of immune cells. Understanding these interactions is vital for deciphering the mechanisms underlying immune modulation in response to SARS-CoV-2 infection and vaccination. Future studies should focus on elucidating these interrelationships to enhance our understanding of their clinical implications in immune assessment and intervention strategies.

In summary, this study enhances the understanding of glycosylation’s profound impact on immune function in the context of COVID-19. By elucidating the relationships between specific glycan profiles (sialylated and high-mannose glycans) and immune status, we provide a foundation for leveraging these markers to improve diagnostic and therapeutic strategies. To fully translate these findings into clinical practice, future research must include longitudinal studies to ascertain causal relationships and monitor the long-term evolution of glycosylation changes, and functional testing to directly investigate how specific glycan modifications affect antibody functionality, binding affinity, and neutralization capacity. This will contribute to a better understanding of immune dynamics and inform more effective clinical interventions for managing the enduring challenges posed by COVID-19.

## 4. Materials and Methods

Serum samples were obtained from 64 patients (49 female, 15 male; mean age 45.2 years) at the Borsod Academic County Hospital (Miskolc, Hungary), categorized into four cohorts (n = 16 each) according to their COVID-19 infection and vaccination status: COVID−Vaccine−, COVID−Vaccine+, COVID+Vaccine−, and COVID+Vaccine+. Post-vaccination samples were collected at a minimum interval of one month after the second Pfizer–BioNTech mRNA dose. The study protocol was approved by the Regional Research Ethics Committee (BORS-02-2021) and adhered to the principles of the Declaration of Helsinki, with written informed consent provided by all participants.

Two datasets were utilized in this analysis:

Glycan Data: This dataset includes various glycan levels measured in individuals categorized by their COVID-19 infection and vaccination status.

Clinical Lab Data: This dataset includes clinical markers such as C-reactive protein (CRP), hemoglobin (Hgb), and vitamin D3 levels, also categorized by infection and vaccination status (Table 1). Both datasets were merged based on the group labels (e.g., COVID−Vaccine−, COVID−Vaccine+, etc.) to enable comprehensive analysis.

The study involved a comparative analysis of glycan markers in serum IgG antibodies among different groups categorized by their COVID-19 infection and vaccination status. Participants included individuals who were vaccinated against COVID-19, those who had recovered from COVID-19, and those who had experienced both vaccination and infection. Serum samples were collected from participants after obtaining informed consent. The samples were processed and stored at −80 °C until analysis to preserve glycan integrity. The glycan profiles of the serum IgG antibodies were analyzed using high-performance liquid chromatography (HPLC) and mass spectrometry (MS) techniques as it has been published in our previous publication [16].

Correlation analyses were conducted to evaluate the relationships between infection status, vaccination status, and glycan levels, treating infection and vaccination as binary variables. Glycan markers that showed significant differences between the groups were evaluated for their potential as biomarkers distinguishing infection-induced immune responses from vaccination-induced responses. The criteria for biomarker candidates included consistent patterns of glycan alteration associated with COVID-19 infection.

## 5. Conclusions

In conclusion, while based on a limited number of samples, this study suggests that specific IgG glycosylation patterns, particularly sialylated and high-mannose structures, could serve as potential biomarkers for differentiating immune responses, which may have important implications for clinical diagnostics and therapeutic strategies. Specifically, these markers could be utilized in diagnostic frameworks to distinguish between natural infection-induced and vaccine-induced immunity, providing a more nuanced molecular-level assessment of a patient’s immune status and memory. Such insights could inform therapeutic strategies by enabling the development of personalized vaccination schedules and the integration of glycan profiling with clinical indicators—such as CRP, hemoglobin, and Vitamin D3 levels—to more accurately monitor vaccine efficacy and disease progression.

Furthermore, exploring the functional implications of specific glycan modifications will enhance our understanding of their roles in antibody efficacy and immune modulation, as these carbohydrate structures are known to dictate the ability of antibodies to bind to specific antigens, activate complement pathways, and interact with various immune cells. For instance, increased sialylation is associated with conferring anti-inflammatory properties and enhancing antibody stability, while variations in high-mannose structures can directly impact the binding affinity and neutralization capacity of the antibody. Although these preliminary findings suggest a significant role for the glycosylation–immune axis in COVID-19, further longitudinal research with larger cohorts is essential to validate these markers and to fully elucidate the mechanisms by which glycan modifications influence long-term clinical outcomes.

## Figures and Tables

**Table 1:** Summary of baseline health indicators across the four patient categories.

Characteristic	COVID−Vaccine−	Std.	COVID−Vaccine+	Std.	COVID+Vaccine−	Std.	COVID+Vaccine+	Std.
Age	46.56	9.51	46.13	10.05	45.19	9.52	42.81	11.69
C-reactive protein	4.09	5.11	1.50	1.72	1.72	2.21	3.64	3.80
White blood cell	7.67	2.04	7.51	2.48	7.53	1.68	6.97	1.51
Hemoglobin	135.94	14.36	133.88	13.68	172.00	109.35	142.13	11.25
Thrombocyte	270.50	63.31	301.00	65.01	280.07	80.63	298.19	57.33
Neutrophil	4.51	1.09	4.66	1.89	37.99	28.20	4.04	1.10
Lymphocyte	2.33	0.93	2.15	0.70	18.54	13.91	2.23	0.48
Eosinophil	0.15	0.08	0.09	0.06	1.23	1.34	0.14	0.07
Vitamin D3	91.84	49.89	89.88	36.94	96.98	42.72	66.64	29.42

## Data Availability

The generated data can be requested from the corresponding author.
